# Shrubs and trees as natural insect protection for grazing animals in Switzerland and the alpine region: A systematic review of in vitro, in vivo and clinical trials

**DOI:** 10.1111/mve.70030

**Published:** 2025-11-19

**Authors:** Theresa Schlittenlacher, Sofie Egli, Michael Walkenhorst, Veronika Maurer

**Affiliations:** ^1^ Research Institute of Organic Agriculture FiBL Frick Switzerland

**Keywords:** biological control, haematophagous Diptera, symbovine flies

## Abstract

Insect infestation in grazing animals is an increasing problem due to factors such as global warming and increasing resistance to insecticides, which have a direct impact on animal health and welfare. According to reports from farmers, observations in zoopharmacognosy and the ingredients of commercial insecticides, various indigenous trees and shrubs with an insecticidal or repellent effect grow or can be planted on pastures. The aim of our systematic review (designed according to the Preferred Reporting Items for Systematic Reviews and Meta‐Analyses (PRISMA) statement) was to identify perennial woody plants from Switzerland and the wider alpine region with a potential to affect the most common dipteran insects that are relevant for grazing animals. Based on a preselection of 399 plants, 114 publications including 403 laboratory and 10 outdoor experiments were found. Essential oils were evaluated most frequently (326 experiments), followed by extracts (118 experiments). Most frequently investigated were the aromatic herbs (Lamiales: Lamiaceae) *Thymus vulgaris* L., *Rosmarinus officinalis* Spenner, *Lavandula angustifolia* Miller, *Salvia officinalis* L., and the trees *Punica granatum* L. (Myrtales: Lythraceae), *Laurus nobilis* L. (Laurales: Lauraceae), *Pinus pinea* L. (Pinales: Pinaceae), *Juniperus communis* L. (Cupressales: Cupressaceae), *Olea europaea* L. (Lamiales: Oleaceae) and *Alnus glutinosa* (L.) Gaertner (Fagales: Betulaceae). These were studied for their effect (repellent, larvicidal, adulticidal) on mosquitoes (Diptera: Culicidae; *Aedes* spp., *Anopheles* spp., *Culex* spp.), and flies (*Musca domestica* L. (Diptera: Muscidae), and *Lucilia sericata* Meigen (Diptera: Calliphoridae)). The results concerning repellent or insecticidal effects provide valuable information about which trees and shrubs should be tested in future outdoor studies involving grazing animals.

## Graphical abstract

- Biting or irritating insects are a threat to the health and welfare of grazing animals.
- This systematic review reveals that several shrubs and bushes growing in the alpine area have insecticidal, insect‐repellent and/or attractant properties.
- The alder *Alnus glutinosa*, juniper *Juniperus communis*, spruce *Picea abies* and walnut *Juglans regia* are promising and easily cultivable trees to be integrated in pastures and agroforestry systems.

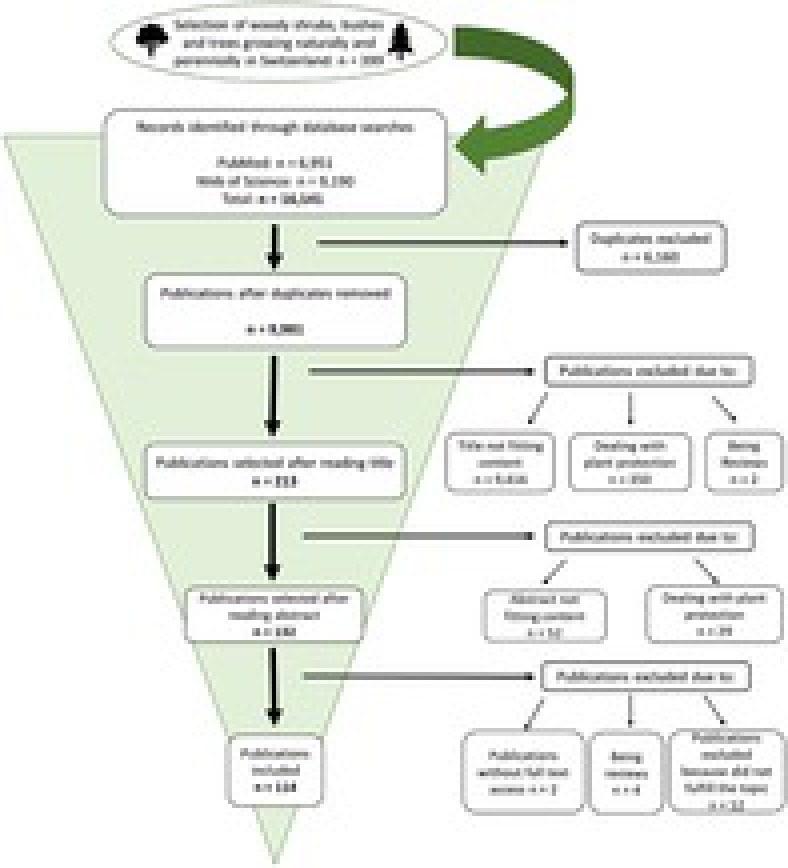

## Zusammenfassung

Der Insektenbefall bei Weidetieren stellt aufgrund von Faktoren wie der globalen Erwärmung und der zunehmenden Resistenz gegen Insektizide ein wachsendes Problem dar, das sich direkt auf die Gesundheit und das Wohlergehen der Tiere auswirkt. Laut Berichten von Landwirten, Beobachtungen in der Zoopharmakognosie sowie den Inhaltsstoffen kommerzieller Insektizide wachsen verschiedene einheimische Bäume und Sträucher mit insektizider oder abwehrender Wirkung auf Weiden oder könnten dort angepflanzt werden. Das Ziel unserer systematischen Übersicht (konzipiert gemäß der PRISMA‐Erklärung) war es, mehrjährige Gehölze aus der Schweiz und dem weiteren Alpenraum zu identifizieren, die das Potenzial haben, die häufigsten für Weidetiere relevanten Insektenarten zu bekämpfen. Auf der Grundlage einer Vorauswahl von 399 Pflanzen wurden 114 Publikationen mit 403 Labor‐ und 10 Freilandversuchen identifiziert. Am häufigsten wurden ätherische Öle (326 Versuche) untersucht, gefolgt von Extrakten (118 Versuche). Am häufigsten untersucht wurden die aromatischen Kräuter (Lamiales: Lamiaceae) *Thymus vulgaris* L., *Rosmarinus officinalis* Spenner, *Lavandula angustifolia* Miller, *Salvia officinalis* L. und die Bäume *Punica granatum* L. (Myrtales: Lythraceae), *Laurus nobilis* L. (Laurales: Lauraceae), *Pinus pinea* L. (Pinales: Pinaceae), *Juniperus communis* L. (Cupressales: Cupressaceae), *Olea europaea* L. (Lamiales: Oleaceae) und *Alnus glutinosa* (L.) Gaertner (Fagales: Betulaceae). Sie wurden auf ihre abwehrende, larvizide und adultizide Wirkung gegenüber Mücken (Diptera: Culicidae; *Aedes* spp., *Anopheles* spp., *Culex* spp.) und Fliegen (*Musca domestica* L. (Diptera: Muscidae) und *Lucilia sericata* Meigen (Diptera: Calliphoridae)) getestet. Die Ergebnisse hinsichtlich der abwehrenden oder insektiziden Wirkung sind für die Auswahl von Bäumen und Sträuchern relevant, die in zukünftigen praktischen Freilandstudien mit Weidetieren genauer untersucht werden sollen.

## INTRODUCTION

In addition to climatic stressors such as heat, grazing animals also are exposed to biting and irritating insects such as midges and flies. Insect exposure increases during the course of the day and as the temperature–humidity index rises (Baldacchino, Puech, et al., 2014; Wardhaugh et al., 2007). As a result of global warming, in addition to the increased direct exposure of animals to heat, an increase in insect infestation is to be expected in the future. The flies, midges and gnats that disturb grazing animals are mainly members of the families Culicidae, Ceratopogonidae, Simuliidae, Tabanidae and Muscidae; ectoparasites that infest grazing livestock also include several species of ixodid ticks (Lysyk, 2011).

Insect infestation has a direct negative impact on animal welfare and the profitability of livestock farming. In dairy cows an increasing number of flies is significantly associated with a lower milk yield and a higher incidence of mastitis (Renčínová et al., 2021; Taylor et al., 2012). In addition, blood‐feeding insects are also significant vectors of disease‐causing pathogens (Baldacchino, Desquesnes, et al., 2014). Emerging or re‐emerging mosquito‐transmitted viral diseases of high importance for livestock are Rift Valley fever (Bunyaviridae), Japanese encephalitis and West Nile fever (Flaviviridae), equine encephalitis (Togaviridae), and the bluetongue virus (Reoviridae). Flystrike is caused by maggots of one of several fly species (e.g. the calliphorid *Lucilia sericata* Meigen). Non‐biting flies play a central role in the intra‐ and interspecific transmission of *Mycoplasma conjunctivae* (Barile (Mycoplasmatales: Mycoplasmataceae)) the primary agent for infective keratoconjunctivitis (pinkeye; Giacometti et al., 2002). Some of these diseases also have zoonotic potential (Cohnstaedt, 2017). Grazing animals are often treated with insecticides or insect‐repellent products. However, their protective effect is unreliable and resistance to such products is constantly increasing. Worldwide studies on insecticide resistance have shown a strong loss of susceptibility of the mosquitoes (Diptera: Culicidae) *Aedes aegypti* L. and *Aedes albopictus* Skuse, amongst others, especially to pyrethroids, one of the most important insecticide classes for vector control (Dusfour et al., 2015; Guo, Hu, et al., 2023). Some active ingredients such as the pyrethroid Deltamethrin in combination with the synergist piperonyl butoxide are highly toxic to bees, fish or earthworms (Basak et al., 2021).

In the field of ethnoveterinary medicine in Europe, there are anecdotal reports from livestock holders that their animals spend more time under certain species of trees when there is increased insect pressure (Schlittenlacher et al., 2022). The hypothesis is that those trees provide a certain degree of protection against insects. However, this could also be random behaviour or uniquely correlated with the seeking of shade. In zoopharmacognosy, studies show that animals specifically use plants as insect repellents in the context of self‐medication. Several studies on European starlings, conducted in North America (Clark & Mason, 1985) and Europe (Gwinner et al., 2000), show that they use fresh plants such as the composites (Asterales: Asteraceae) *Achillea millefolium* L. and *Solidago* spp. L., *Daucus carota* L. (Apiales: Apiaceae), and *Sambucus nigra* L. (Dipsacales: Adoxaceae) as nesting material, even if these plants are not directly available in the neighbourhood. These plant species contain essential oils that have been shown to affect arthropods in high concentrations (e.g. Pavela et al., 2018).

Therefore, the purpose of this systematic review was to gain information about potential insecticidal, attractant or repellent activities of woody shrubs and trees in laboratory and outdoor studies. The focus was on the native or regionally cultivable flora and on the most common disturbing and blood‐feeding dipteran insects of Switzerland and the alpine region. This could provide indications of whether there might be a benefit of cultivating such plants as natural shelters for grazing animals, in particular trees in agroforestry systems.

## MATERIALS AND METHODS

The recommendation of the PRISMA statement (Liberati et al., 2009; Page et al., 2021) and the AMSTAR measurement tool (Shea et al., 2007, 2017) served as the basis for the design of the systematic review. The research question was formulated based on the PICOS scheme (Moher et al., 2009):

The **population** being addressed consisted of disturbing and blood‐feeding dipteran species associated with grazing animals, and the **intervention** was a treatment with raw material or extracts based on woody shrubs, bushes and trees growing naturally and perennially in Switzerland. The **comparator** was either no treatment or a standard treatment (without plant‐based material). The **outcome** was the effect of the plant material or the extract of the woody shrubs, bushes and trees on insect development, reproduction, survival and/or as a repellent. The **study designs** included in vitro, in vivo and clinical data.

### Relevance screening and selection of plant species

To choose eligible plant species, two different initial sources were screened during June and July 2023: Infoflora (Infoflora, 2015) and Flora Helvetica app (Lauber et al., 2018). The flora of Switzerland can be considered as representative for the European alpine flora, given that it covers northern and southern alpine and sub‐alpine slopes and all altitudes. The search included gymnosperms and woody angiosperms, which were classified by life form type—phanerophytes, woody chamaephytes, and lianas—to verify that tree and shrub species met the criteria of being woody, perennial and indigenous or cultivable in Switzerland. Subspecies were not recorded in detail. Neophytes were included unless they are considered as invasive or potentially invasive species (Infoflora, 2023) or are distributed only in one biogeographical region according to the Infoflora base map (Infoflora, 2015). The information on each plant, including its common name (compared under Kew Science (Kew, 2023)), family affiliation, growth form, distribution and indigeneity was recorded in tabular form (Supplementary File 1).

### Selection of scientific references

#### Bibliographic search

Scientific information on these plant species was searched in PubMed (“PubMed.Gov,” n.d.) and Web of Science (WebOfScience, 2020). Both bibliographic sources were consulted in the time between 27 July 2023 and 18 August 2023. No specific timeframe of publication years and no general refinement by keyword research were considered. First, we created a search term which consisted of the Latin name and, if applicable, the English name of the respective plant and insect species. As we used the term musc*, we explicitly excluded all search results for “muscles”. This led to the following search term: (“Name lat” OR “Name engl”) AND (“symbovine flies” OR tabanid* OR tabanus OR musc* OR stomox* OR blowfl* OR calliphor* OR haematobia OR simuliid* OR nematocera OR gnat* OR mosquito* OR fly OR midge) NOT muscle*.

For the two Ericaceae species (Ericales: Ericaceae) *Calluna vulgaris* Salisbury and *Erica tetralix* L., the search for authors was removed from the refined results in Web of Science, and PubMed was searched exclusively by title and abstract, as the English plant name “heather” or “heath*” led to search results with the author name “Heather”. For hybrid species (e.g. *Populus x canescens*), a second search was carried out without the “x” in the name to ensure that all common spellings were captured.

Publications found in Web of Science and publications found in PubMed were recorded in one folder each in an Mendeley database (Elsevier, 2024).

#### Removing duplicates automatically and manually

The two folders were subsequently merged and all duplicates deleted. Duplicates that were not automatically detected were removed manually afterwards.

#### Definition of inclusion and exclusion criteria

The inclusion and exclusion criteria were predefined by three scientists. Only peer‐reviewed publications with an abstract written in English or in one of the Swiss national languages (German, French, Italian) were considered for further evaluation. Publications were included if the title contained one of the plants listed in Supplementary File 1 in relation to an effect (regardless of the type: positive or negative) on insect species from the search term. Either the botanical or the English name or both names could be mentioned in the title or only the corresponding plant family. Publications were included if these plants were investigated individually or as part of a mixture with defined composition. Mixtures were allocated to the plant that belonged to the eligible plant list (Supplementary File 1); where several plants of a mixture were eligible (e.g. in 1:1 a mixture of *Pinus nigra* Arnold (Pinales: Pinaceae) and *Satureja montana*L. (Lamiales: Lamiaceae) (Benelli et al., 2017)), the experiment was allocated to both plants. Excluded were publications in which (1) the plant was not investigated as a whole (only as a single chemical component) or (2) the full text could not be accessed. Publications that dealt purely with insect pests of plants and thus belonged to the category of plant protection were excluded. In addition, reviews as well as abstracts of conference contributions without full text contributions were excluded.

#### Refining with manual title and abstract check

First, the titles of the publications were screened manually by a first evaluator and an inclusion or exclusion for the systematic review was made according to the predefined inclusion and exclusion criteria described above. The next step was to screen the abstracts of the references included after the manual screening of the titles, applying the same criteria as for the title screening. These publications were then included or excluded again. A small sample of randomly selected publications (approximately 5%) was additionally screened by another evaluator to confirm the decisions of the first evaluator. If the title or abstract was not sufficiently informative for decision‐making, the full text was consulted. For these cases, a final decision was made in accordance with the three evaluators.

### Classification of the publications (including one or more experiments)

The included studies that complied with the study designs were either laboratory (mainly bioassays) or outdoor studies, both with or without host. Before adding the information of the included publications in a results table, a distinction between “publication” (as one scientific publication) and “experiment” was made, based on the fact, that some publications reported results of several experiments or of experiments with more than one plant species. Other publications referred to more than one insect species or developmental stage of the insect (e.g. *Punica granatum* L. (Myrtales: Lythraceae) tested in adult female *Culex gelidus* Theobald (Diptera: Culicidae), adult female *Culex quinquefasciatus* Say (Diptera: Culicidae) and in 4th instar larvae of *Cx. quinquefasciatus*; (Kamaraj et al., 2010)).

Therefore, the following definition of “experiment” was used: Experiment = plant species × insect species × insect developmental stage × target of trial × publication. Hence, as an example, a publication referring to three controlled trials with one plant species (*Thymus vulgaris* L. (Lamiales: Lamiaceae)), one insect species (*A. aegypti*) and three targets of trials (larvicidal activity, adulticidal activity, repellency) would lead to three experiments: *T. vulgaris* × larvicidal activity on *A. aegypti*, *T. vulgaris* × adulticidal activity on *A. aegypti*, and *T. vulgaris* × repellent activity on *A. aegypti* (de Oliveira et al., 2021). The same was applicable when a plant species was tested in different combinations with other plant species in a publication. For instance, *Salvia officinalis* L. (Lamiales: Lamiaceae) × *Citrus aurantium* L. (Sapindales: Rutaceae) × adult *Anopheles dirus* Peyton & Harrison (Diptera: Culicidae), *S. officinalis* × *Mentha piperita* L. (Lamiales: Lamiaceae) × adult *A. dirus*, *S. officinalis* × *Pimpinella anisum* L. (Apiales: Apiaceae) × adult *A. dirus* led to three experiments (Sutthanont et al., 2022).

### Assessment of the experiments

All experiments were evaluated considering the following information on (1) **plants**: species, family, pharmaceutical form of the plant preparation used, dosage/concentration, mixture or single plant preparation, (2) **insects**: species, developmental stage and target of the study, (3) **method** of the experiment: trial specification, number of insects included, duration of exposure, comparator, categorization into laboratory or outdoor, with and without host, and (4) **outcomes** of the experiment. Data were summarized in a data table (Supplementary File 2, sheet “Experiments”).

For each investigated effect the outcomes were categorized as follows: attractant, repellent, oviposition deterrence or zero effect, as well as larvicidal, adulticidal, ovicidal, pupicidal or zero effect. Effects that could not be assigned to a category were considered “not relevant” (nr). The scoring system was based on the information/statements given in the publication. It was assessed whether a plant substance had an effect on the insect species or not. Positive effects were rated with “+” e.g. “a+” for a positive adulticidal effect, whereas no effects were rated with “0” e.g. “a0” for no adulticidal effect. This was determined in most experiments with a positive and/or negative control group, which meant that non‐significant differences were rated with 0, and significant positive effects were rated with a “+”, without further gradations. The details of the effect ratings for each experiment can be found in Supplementary File 2.

## RESULTS

### Procedure of systematic literature review

**FIGURE 1:**
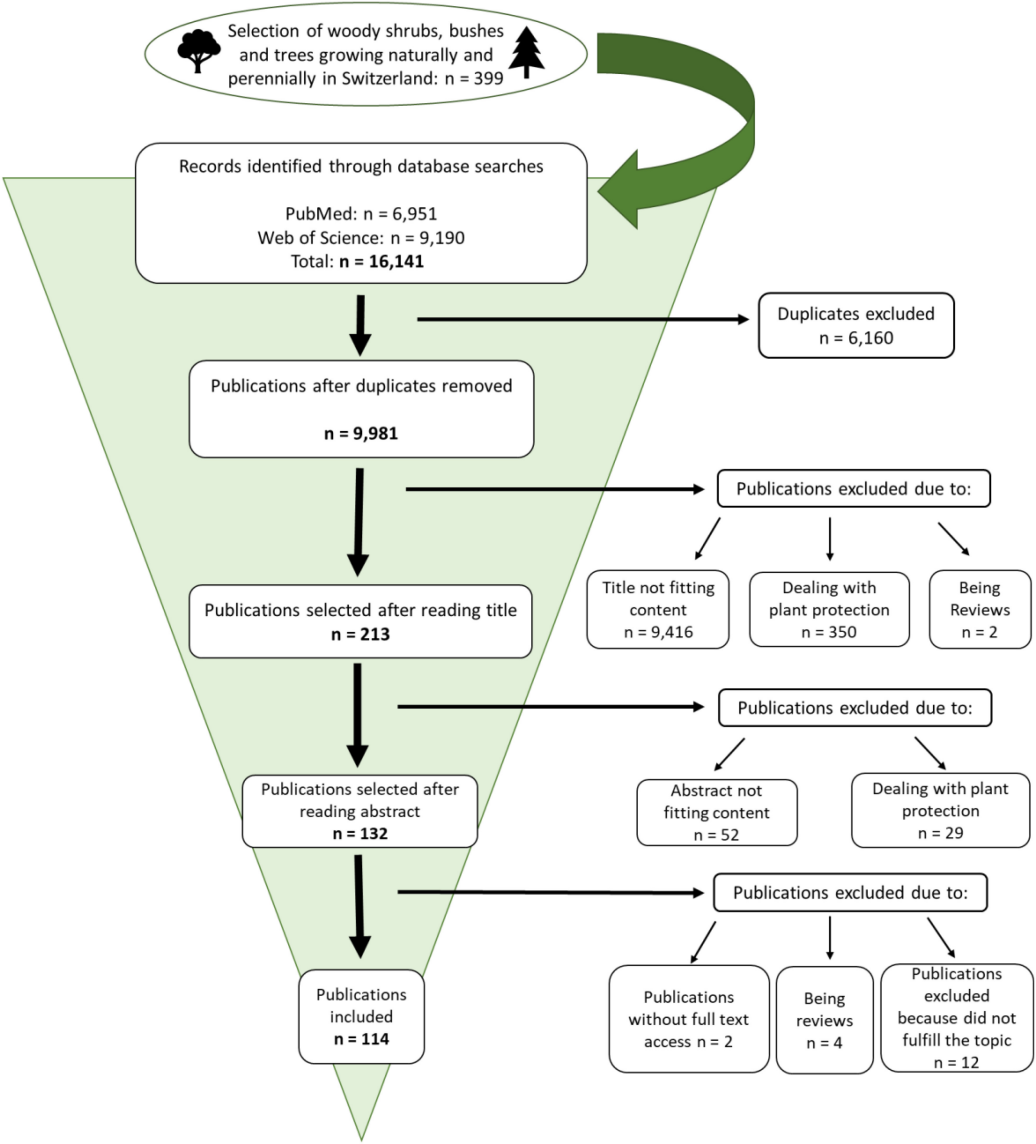
Process of the systematic literature review on shrubs and trees as natural insect protection for grazing animals in Switzerland and the alpine region.

Database screening resulted in 16,141 hits, and 9981 publications remained after the removal of 6160 duplicates. After screening the titles, 9768 publications were excluded because they did not match the defined criteria; these included 279 publications dealing with insect pests of crops, thus falling under the category of plant protection (Figure 1).

Finally, a total of 213 publications remained. After reading the full text, 114 publications were selected (Figure 1) and summarized (Supplementary File 2).

The studies considered in our review were published between 1999 and 2023. We observed that there was, in general, an increasing number of publications and experiments from year to year.

### Frequencies of published evidence

#### Experiments and their targets

The 114 publications found in our search included a total of 503 experiments. According to our definition key, each publication comprised between 1 (e.g. Chung et al., 2011) and 34 experiments (e.g. Traboulsi et al., 2005).

**FIGURE 2:**
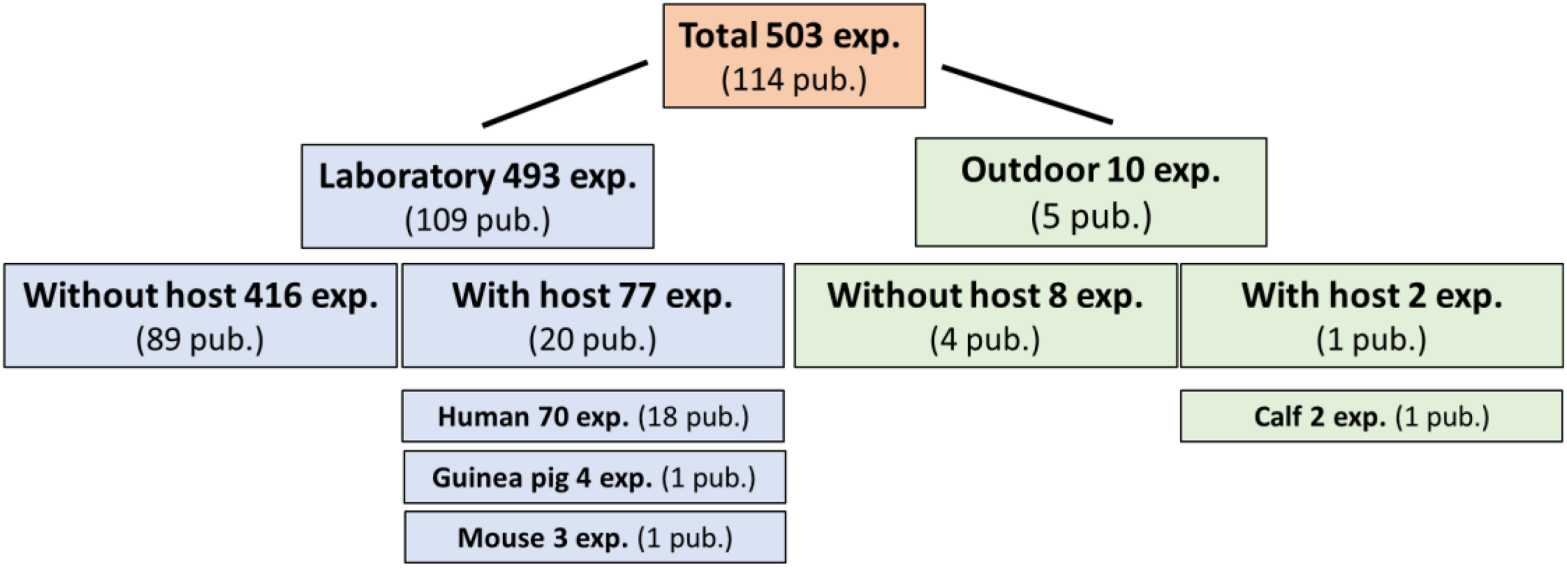
Types of experiments found in the systematic review on shrubs and trees as natural insect protection for grazing animals in Switzerland and the alpine region. Exp. = experiments, pub. = publications.

The experiments were divided into two groups, namely “laboratory experiments” and “outdoor experiments” (Figure 2). The majority (493 experiments from 109 publications) were laboratory experiments, including 416 experiments without a host, and 77 with a host (human volunteers, guinea pigs or mice). In addition, 10 experiments were categorized as outdoor, including two experiments with a host (calves).

Regarding the 503 experiments included in our systematic review, 213 experiments were on larvicidal activity and larval development (including six on pupicidal activity) and 73 experiments targeted adult longevity and adulticidal activity. There were 33 experiments on oviposition deterrence and ovicidal activity and 127 experiments on repellency properties (three of them on repellency as well as larvicidal activities). The other 57 experiments varied in their objectives and had very specific targets, such as acetylcholinesterase inhibition, ingestion toxicity or mitochondrial enzyme alteration in the insects (Supplementary File 2).

#### Plant species studied in the experiments

A total of 399 trees, shrubs and bushes were included in the preselection of plants relevant to Switzerland (according to Flora (2015) and Lauber et al. (2018)). In our systematic literature search we found results for 47 plants according to our preselection list (Supplementary File 1). In ten cases only the English common name (cypress, juniper, lavender, pine, rose, rosemary, sage, thyme, thyme red, thyme white; six publications) or the genus (*Juniperus* sp.; one publication) was given. Where only one species of the same genus appeared in our search, these records were pooled (e.g. “juniper” and “*Juniperus* sp.” with *Juniperus communis* L. (Cupressales: Cupressaceae)) while they were treated separately in the case of “pine”, because several *Pinus* spp. (Pinales: Pinaceae) appeared in the search (*Pinus nigra* Arnold, *Pinus pinea* L., *Pinus strobus* L.). See Table 1 for details.

**TABLE 1:** Plant species with insecticidal, insect repellent and/or attractant properties according to the systematic review with their used plant parts and pharmaceutical form (plant species sorted according to the number of publications and experiments).

Plant species	Plant part used	Pharmaceutical form	Number of publications/experiments	References
*Rosmarinus officinalis* Spenner (Lamiaceae)	Na, stem, shoot, aerial parts, leaves, tops	Essential oil, ethanol extract, methanol extract, biological hydrolate	38/72 (including one only giving the common name “Rosemary”)	Adams et al. (2016), Ahmadi et al. (2022), Alavez‐Rosas et al. (2022), Al‐Massarani et al. (2019), Amer and Mehlhorn (2006b), Jeno et al. (2022), Choi et al. (2002), Cilek et al. (2011), Conti et al. (2010), Deletre et al. (2013), Drapeau et al. (2009), Bosly and Bosly (2022), Fadl et al. (2022), Freitas et al. (2010), Giatropoulos et al. (2018), Gillij et al. (2008), Govindarajan (2011), Hasaballah (2015), Hazarika et al. (2020), Hieu et al. (2010), Kang et al. (2009), Khater et al. (2009), Khater et al. (2011, 2023), Kröber et al. (2018), Kulma et al. (2018), Pavela (2009), Prajapati et al. (2005), Ramzi et al. (2022), Sheng et al. (2020), Sutthanont et al. (2022), Wu et al. (2022), Yu et al. (2013), Vatandoost et al. (2019), Waliwitiya et al. (2009), Warikoo et al. (2011), Wu et al. (2013), Zeghib et al. (2020)
*Thymus vulgaris* L. (Lamiaceae)	Na, aerial parts, flowering tops, whole plant, leaves	Essential oil, ether extract, methanol extract, acetone extract, lotion, chitosan encapsulation of thyme oil nanoemulsion, co_2_ extract, hexane extract	32/84 (including 8 only giving the common names: 1 “Red Thyme”, 1 “White Thyme”, 6 “Thyme”)	Ahmadi et al. (2022), Baz et al. (2022), Bouguerra et al. (2018), Bangonan et al. (2022), Barnard (1999), Choi et al. (2002), De La Torre Rodriguez et al. (2013), de Oliveira et al. (2021), Deletre et al. (2013), Drapeau et al. (2009), El Zayyat et al. (2017), Fadl et al. (2022), Giatropoulos et al. (2018), Girgenti and Suss (2002), Gupta et al. (2022), Hieu et al. (2010), Kang et al. (2009), Kazempour et al. (2021), Li et al. (2023), Pavela (2007b), Pavela et al. (2009), Pérez López et al. (2015), Rios et al. (2017), Sajfrtova et al. (2013), Sheng et al. (2020), Showler and Harlien (2022), Wu, Zhang, and Yang (2019), Wu et al. (2022), Vatandoost et al. (2019), Zhu and Zeng (2006)
*Lavandula angustifolia* Miller (Lamiaceae)	Na, leaves, flowering tops	Methanol extract, esssential oil, ethanol extract, co_2_ extract, hexane extract	22/60 (including one only giving the common name “Lavender”)	Adams et al. (2016), Ahmadi et al. (2022), Al‐Massarani et al. (2019), Amer and Mehlhorn (2006a), Bedini et al. 2019, Conti et al. (2010), El‐Akhal et al. (2021), Bosly and Bosly and Bosly (2022), Giatropoulos et al. (2018), Guo, Luo, et al. (2023), Hazarika et al. (2020), Kang et al. (2009), Khater et al. (2018), Pavela (2009), Sajfrtova et al. (2013), Sheng et al. (2020), Sinthusiri and Soonwera (2013), Wu et al. (2013), Choi et al. (2002), Girgenti and Suss (2002), Hieu et al. (2010), Krcmar and Gvozdic (2016)
*Laurus nobilis* L. (Lauraceae)	Leaves, na	Essential oil	12/33	Aissaoui et al. (2023), Andrade‐Ochoa et al. (2018), Bouzidi et al. (2020), Erler et al. (2006), Mariano Fernandez et al. (2018), Pavela et al. (2009), Sheng et al. (2020), Tabanca et al. (2013), Traboulsi et al. (2005), Wu, Kang, et al. (2019), Wu et al. (2022), Zarenezhad et al. (2022)
*Salvia officinalis* L. (Lamiaceae)	Aerial part, na	Essential oil	9/29 (including one only giving the common name “Sage”)	Ali et al. (2017), Baz et al. (2022), Castillo‐Morales and Duque (2020), Girgenti and Suss (2002), Kang et al. (2009), Pavela (2007a), Rios et al. (2017), Sutthanont et al. (2022), Castillo‐Morales et al. (2019)
*Juniperus communis* L. (Cupressaceae)	Leaves, na, bark, twigs, fruit	Essential oil	9/16 (including one only giving the common name “Juniper” and one naming the genus “Juniperus”)	Amer and Mehlhorn (2006b), Carroll et al. (2011), Kröber et al. (2018), Levchenko et al. (2021), Pavela (2007a), Pavela et al. (2021), Sheng et al. (2020), Stappen et al. (2018), Wu et al. (2022)
*Punica granatum* L. (Lythraceae)	Fruit rind, na	Ethanol extract, methanol extract, ethyl acetat extract, hexane extract, chloroform extract, acetone extract	7/58	Al‐Azab et al. (2020), Al‐Massarani et al. (2019), Baranitharan et al. (2019), Hamad et al. (2019), Jebanesan et al. (2021), Kamaraj and Rahuman (2010), Taher et al. (2012)
*Olea europaea* L. (Oleaceae)	Na, fruit, leaves	Oil, essential oil, smoke	5/14	Amer and Mehlhorn (2006a), Dube et al. (2011), Karunamoorthi et al. (2009), Njoroge and Berenbaum (2019), Olbamo et al. (2021), Sheng et al. (2020)
*Cupressus sempervirens* L. (Cupressaceae)	Leaves, aerial parts, branches, na	Essential oil, nanoemulsion	5/11 (including one only giving the common name” Cypress”)	Almadiy and Nenaah (2022), Baz et al. (2022), Drapeau et al. (2009), Giatropoulos et al. (2013), Wu et al. (2022)
*Satureja montana* L. (Lamiaceae)	Flowering aerial parts, leaves, na	Essential oil	3/8	Benelli et al. (2017), Deletre et al. (2013), Hieu et al. (2010)
*Pinus sylvestris* L. (Pinaceae)	Needles, twigs, na	Essential oil	3/7	Fayemiwo et al. (2014), Levchenko et al. (2021), Pavela et al. (2021)
*Juglans regia* L. (Juglandaceae)	Male flower, leaves	Methanol extract, essential oil	3/5	Ali et al. (2019), Köroǧlu et al. (2016), Kulma et al. (2018)
*Ginkgo biloba* L. (Ginkgoaceae)	Leaves, exocarp	Ethanol extract, methanol extract, dichloromethane extract, silver nanoparticles with plant extract, essential oil	3/5	Ahmad et al. (2011), Balashanmugam et al. (2018), Sun et al. (2006)
*Hyssopus officinalis* L. (Lamiaceae)	Stem, leaves, flowering aerial parts	Essential oil, methanol extract, ethanol extract	3/4	Al‐Massarani et al. (2019), Benelli et al. (2017), Pavela (2007a)
*Vitis vinifera* L. (Vitaceae)	Na, fruit	Methanol extract, essential oil, ethanol extract	3/4	Al‐Massarani et al. (2019), Khater et al. (2023), Taher et al. (2012)
*Picea abies* (L.) Hermann Karsten (Pinaceae)	Needles, twigs	Essential oil	2/5	Levchenko et al. (2021), Pavela et al. (2021)
*Pinus nigra* Arnold (Pinaceae)	Needles, twigs with needles	Essential oil	2/4	Benelli et al. (2017), Koutsaviti et al. (2015)
*Chamaecyparis lawsoniana* (A. Murray) Parlatore (Cupressaceae)	Wood shavings, aerial parts	Essential oil	2/3	Dolan et al. (2007), Giatropoulos et al. (2013)
*Pinus* sp. (only common name “Pine” available) L. (Pinaceae)	Na	Essential oil	2/2	Wu, Zhang, and Yang (2019), Wu et al. (2022)
*Ficus carica* L. (Moraceae)	Sap, seed pods/fruit	Milky sap extract, water suspension	2/2	Chung et al. (2011), Müller et al. (2011)
*Pinus pinea* L. (Pinaceae)	Leaves	Essential oil	1/17	Traboulsi et al. (2005)
*Alnus glutinosa* (L.) Gaertner (Betulaceae)	Leaves	Fresh plant	1/11	David et al. (2000)
*Populus nigra* L. (Salicaceae)	Leaf litter, fresh leaves	Water suspension	1/6	David et al. (2000)
*Quercus robur* L. (Fagaceae)	Leave litter, fresh leaves	Water suspension	1/6	David et al. (2000)
*Morus alba* L. (Moraceae)	Leaves	Petroleum ether extract	1/4	Srivastava et al. (2023)
*Sambuccus ebulus* L. (Adoxaceae)	Leaves, fruit	Methanol extract, ethanol extract	1/4	Motevalli‐Haghi et al. (2020)
*Pinus strobus* L. (Moraceae)	Needles	Essential oil	1/2	Koutsaviti et al. (2015)
*Platycladus orientalis* (L.) Franco (Cupressaceae)	Leaves	Essential oil	1/2	Sanei‐Dehkordi et al. (2018)
*Teucrium montanum* L. (Lamiaceae)	Aerial parts	Essential oil	1/2	Pavela et al. (2020)
*Thuja occidentalis* L. (Cupressaceae)	Na	Essential oil	1/2	Barnard (1999)

*Note*: The table shows number of individual experiments and the related publications. Plant species identified in the systematic review with one experiment in one publication only: *Abies alba*, essential oil (Levchenko et al., 2021); *Acer campestre*, methanol extract (Pavela, 2007a); *Acer negundo*, methanol extract (Pavela, 2007a); *Acer platanoides*, methanol extract (Pavela, 2007a); *Acer pseudoplatanus*, methanol extract (Pavela, 2007a); *Fraxinus excelsior*, methanol extract (Pavela, 2007a); *Fumana ericoides*, methanol extract (Pavela, 2007a); *Humulus lupulus*, essential oil (Bedini et al., 2016); *Jasminum officinale*, essential oil (Hazarika et al., 2020); *Juglans nigra*, oil (Njoroge & Berenbaum, 2019); *Laburnum anagyroides*, methanol extract (Pavela, 2007a); *Lonicera caprifolium*, essential oil (Muturi et al., 2019); *Prunus persica*, methanol extract (Seo & Park, 2012); *Prunus dulcis*, essential oil (Baz et al., 2022); *Pyracantha coccinea*, methanol extract (Koc et al., 2016); Rose (only common name given), essential oil (Wu et al., 2022); *Vitis labrusca*, oil (Njoroge & Berenbaum, 2019).

Abbreviation: na, no information available.

#### Pharmaceutical form of the plants used

Table 1 summarizes the plant species described in the experiments with their used plant parts and pharmaceutical form. Details are presented in Supplementary File 2. In the 503 experiments, essential oils of plants were used most frequently (326 experiments), followed by extracts (118 experiments) that were produced using various solvents (five acetone extracts, 10 chloroform extracts, four CO_2_ extracts, one dichloromethane extract, 17 ethanol extracts, one ether extract, 14 ethyl acetate extracts, 19 hexane extracts, 40 methanol extracts, six petroleum ether extracts, one toluene extract). Extracts of volatiles used in six experiments were produced using undefined methods and can therefore not be allocated to “essential oils” or “extracts”. The plant was used directly in 35 experiments, either as a whole plant soaked in water (26 experiments), as direct plant oil (five experiments), as smoked plant parts (three experiments), or as sap (one experiment). Further 18 experiments investigated other pharmaceutical forms (seven nano emulsions, five creams or lotions, three biological hydrolats, two leachates, one silver nanoparticle).

In 428 experiments, the plant or the plant extract alone was examined. In 75 experiments (10 publications), the target plant was either part of a mixture with one or more plants (e.g. in a commercial product including essential oils of *Rosmarinus officinalis* Spenner (Lamiales: Lamiaceae), *Cinnamomum verum* Presl (Laurales: Lauraceae) and *Cymbopogon schoenanthus* (L.) Sprengel (Poales: Poaceae); (Cilek et al., 2011)) or it was analysed both individually and in a mixture with at least one other plant (e.g. repellent activity of 10 essential oils including *S. officinalis* alone as well as the combination of *S. officinalis* with *Ocimum basilicum* L. (Lamiales: Lamiaceae) by (Sutthanont et al., 2022)). Details can be found in Supplementary File 2.

#### Insect species studied in the experiments

The 503 experiments investigated the effect of plant substances and preparations on a range of blood‐feeding and irritating dipteran species from a total of seven families (Calliphoridae, Ceratopogonidae, Chironomidae, Culicidae, Muscidae, Simuliidae and Tabanidae). Effects on the mosquitoes (Diptera: Culicidae) *A. aegypti* (103 experiments), *Culex pipiens* L. (88 experiments) and *Cx. quinquefasciatus* (87 experiments) were analysed most frequently, followed by *A. albopictus* (45 experiments), *Musca domestica* L. (Diptera: Muscidae) (40 experiments), *Anopheles stephensi* Liston (Diptera: Culicidae) (38 experiments), *L. sericata* (28 experiments) and *Anopheles gambiae* Giles (Diptera: Culicidae) (11 experiments). Experiments with a total of 25 insect species were analysed, plus two experiments where only information on the genus was provided (Table 2).

**TABLE 2:** Blood‐feeding and annoying dipteran species (Nematocera and Brachycera) investigated in the systematic review on insecticidal, insect repellent and/or attractant properties of shrubs and trees growing in the Alpine region.

Suborder	Family	Genus	Species	Number of experiments
Nematocera	Ceratopogonidae	*Forcipomyia*	*Forcipomyia taiwana* (Tokuichi Shiraki)	1
	Chironomidae	*Chironomus*	*Chironomus annularius* Kieffer	4
	Culicidae	*Aedes*	*Aedes aegypti* L.	104
			*Aedes albopictus* Skuse	45
			*Aedes rusticus* (Rossi)	4
		*Anopheles*	Not specified	2
			*Anopheles albimanus* C. R. G. Wiedemann	3
			*Anopheles arabiensis* Patton	4
			*Anopheles dirus* Peyton & Harrison	6
			*Anopheles gambiae* Giles	11
			*Anopheles quadrimaculatus* Say	4
			*Anopheles stephensi* Liston	38
			*Anopheles subpictus* (Grassi)	2
		*Culex*	Not specified	1
			*Culex gelidus* Theobald	6
			*Culex pipiens* L.	88
			*Culex quinquefasciatus* Say	87
			*Culex tritaeniorhynchus* Giles	4
		*Culiseta*	*Culiseta longiareolata* Macquart	3
Brachycera	Calliphoridae	*Lucilia*	*Lucilia sericata* Meigen	28
	Muscidae	*Haematobia*	*Haematobia irritans irritans* (L.)	4
		*Musca*	*Musca domestica* L.	40
		*Stomoxys*	*Stomoxys calcitrans* (L.)	7
	Simuliidae	*Simulium*	*Simulium variegatum* Meigen	4
	Tabanidae	*Haematopota*	*Haematopota pluvialis* L.	1
		*Tabanus*	*Tabanus bromius* L.	1
			*Tabanus tergestinus* Egger	1
Total number of experiments				503

### Outcome of published evidence

#### Outcome of the most often investigated plant and insect species

The ten plant species *T. vulgaris*, *R. officinalis*, *Lavandula angustifolia* Miller (Lamiales: Lamiaceae), *P. granatum*, *Laurus nobilis* L. (Laurales: Lauraceae), *S. officinalis*, *P. pinea*, *Juniperus communis* L. (Cupressales: Cupressaceae), *Olea europaea* L. (Lamiales: Oleaceae), and *Alnus glutinosa* (L.) Gaertner (Fagales: Betulaceae) were mentioned at least five (and up to 69) times. A total of 339 experiments dealt with their effect on one of the eight insect species for which at least seven (and up to 81) experiments were found in the systematic review (Table 3).

**TABLE 3:** Assessment of effects^a^ of the plant species mentioned in at least five publications on the eight most frequently mentioned insect species identified in the systematic review on insecticidal, insect repellent and/or attractant properties of shrubs and trees growing in the alpine region.

Plant species	Number of publications	Insect species								Total
		*Aedes aegypti*	*Aedes albopictus*	*Anopheles gambiae*	*Anopheles stephensi*	*Culex pipiens*	*Culex quinquefasciatus*	*Lucilia sericata*	Musca domestica	
***Rosmarinus officinalis***	**38**	**21** (7 r+/1 r0/3 op+/6 L+/1 a+/1 a0/1 o+/1o0)	**7** (3 r+/3 l+/1 a+)	**4** (2 r0/1 a+/1 a0)	**6** (1 od+/1 r+/2 L+/1 a+/1 o0)	**15** (2 r+/10 L+/3 a+)	**10** (2 r+/1 od+/3 L+/1 L0/2 a+/1 o0)	**1** (1 l+)	**2** (1 r+/1 a+)	**66** (16 r+/3 r0/3 op+/2 od+/25 L+/1 l0/10 a+/2 a0/1 o+/3 o0)
***Thymus vulgaris***	**30**	**16** (5 r+/1 r0/8 L+/2 a+)	**8** (4 r+/4 l+)	**3** (1 r+/1 a+/1 irritation)	**4** (4 l+)	**9** (1 r+/5 l+/3 a+)	**15** (2 od+/10 l+/3 a+)	No experiments	**16** (4 L+/2 l0/8 a+/2 a0)	**71** (11 r+/1 r0/2 od+/35 L+/2 l0/17 a+/2 a0/1 irritation)
***Lavandula angustifolia***	**22**	**5** (1 r+/1 r0/2 l+/1 a0)	**5** (2 r+/2 l+/1 l0)	No experiments	No experiments	**5** (1 r+/3 l+/1 a+)	**2** (2 l+)	**27** (3 r+/3 r0/4 od+/9 L + & p+/3 l0 & p0/4 a+/1 o+)	**9** (1 r+/1 od+/1 o0/4 a+/2 a0)	**53** (8 r+/4 r0/5 od+/1 o0/9 l+/9 L + & p+/3 l0 & p0/1 l0/9 a+/1 o+/3 a0)
***Laurus nobilis***	**12**	**4** (2 r+/1 l+/1 l0)	**3** (2 r+/1 l0)	No experiments	**2** (2 l+)	**3** (1 r+/2 L+)	**3** (2 L+/1 p+)	No experiments	No experiments	**15** (5 r+/7 l+/2 l0/1 p+)
***Salvia officinalis***	**9**	**15** (6 r+/1 od+/3 l+/1 o+/1 p+/1 a+/2 others)	No experiments	No experiments	No experiments	**2** (1 l+/1 a+)	**2** (1 r+/1 l+)	No experiments	No experiments	**19** (7 r+/5 l+/1 od+/1 o+/1 p+/2 a+/2 others)
***Juniperus communis***	**9**	**6** (2 r+/3 l+/1 a0)	**2** (1 r+/1 l+)	No experiments	**1** (1 l+)	No experiments	**3** (3 l+)	No experiments	**3** (3 a+)	**15** (3 r+/8 l+/3 a+/1 a0)
***Punica granatum***	**7**	**4** (2 l+/1 p+/1 a+)	**1** (1 att+)	No experiments	**20** (8 r+/8 L+/4 o+)	**2** (2 l+)	**25** (7 r+/11 L+/4 o+/3 a+)	No experiments	No experiments	**52** (15 r+/1 att+/23 L+/1 p+/8 o+/4 a+)
***Olea europaea***	**5**	**7** (1 r+/2 r0/1 od+/2 l+/1 l1 and p+)	**1** (1 l0)	No experiments	No experiments	No experiments	No experiments	No experiments	No experiments	**8** (1 r+/2 r0/1 od+/2 l+/1 l + and p+/1 l0)
***Cupressus sempervirens***	**5**	**1** (1 r+)	**3 (**1 r+/1 r0/1 l +)	No experiments	No experiments	**1** (1 l+)	**6 (**2 r+/2 a+/2 l +)	No experiments	No experiments	**11** (4 r+/1 r0/4 l + /2 a+)
**Total number of experiments**		**79**	**30**	**7**	**33**	**36**	**62**	**28**	**30**	**305**

*Note*: The bold values indicates the total number of publications.

^a^ Effects: explanation of the abbreviations: r + = repellent effect; r0 = zero repellent effect; att + = attractive effect; att0 = no attraction; od + = effect on oviposition deterrence; od0 = zero effect no oviposition deterrence; l + = larvicidal effect; l0 = no larvicidal effect; a + = adulticidal effect; a0 = no adulticidal effect; p + = pupicidal effect; p0 = no pupicidal effect; o + = ovicidal effect; o0 = no ovicidal effect; others = mitochondrial effect and effect on the DNA; irritation = stop of the experiment due to contact irritation of the host's skin;

The repellent effect was tested in 82 experiments, of which a repellent effect (of varying intensity and duration, r+) was demonstrated in 72 experiments, and no repellent effect occurred in 10 experiments (r0). A larvicidal effect (l+) was demonstrated in 159 experiments while 12 experiments revealed no larvicidal effect (l0). An adulticidal effect (a+) could be demonstrated in 46 experiments, whereas, eight experiments showed no adulticidal effect (a0). Some individual experiments focused on attraction, oviposition deterrence, ovicidal or pupicidal effects (Table 3).

#### Outcome of laboratory experiments with a host

Our literature search yielded a total of 19 publications describing 77 laboratory experiments with a host (Figure 2); results are summarized in Table 4. Most frequently, repellency bioassays were performed with a human arm/hand as the host, analyzing the landing and biting rate of insects. Two publications report on experiments with laboratory animals (guinea pigs and mice). Several extracts, or essential oils, had a repellent effect on *Aedes* spp. and/or other target insects. All these studies had a negative and/or positive control group (details can be found in Supplementary File 2).

**TABLE 4:** Summary of laboratory experiments with a host identified in the systematic review on insecticidal, insect repellent and/or attractant properties of shrubs and trees growing in the alpine region (10 most frequently mentioned plants are highlighted in bold).

Publication	Study design	Host	Exposure	Plant species	Pharmaceutical form	Concentration	Effect on target insect	Effect description
Alavez‐Rosas et al. (2022)	Repellence bioassay: human bait technique	Human hand	5 min	***Rosmarinus officinalis***	Essential oil	3%, 3,5% and 10%	r + on adult *Aedes aegypti*	EO significantly more repellent than negative control, median percentage between 60% and 94% in combination with *Mentha arvensis*, *Cymbopogon nardus* and *Eugenia caryophyllata*
Baranitharan et al. (2019)	Repellency test—(25 cm^3^ of skin exposed), the other arm is untreated and serves as control	Human arm	Up to 240 min	***Punica granatum***	Ethanol extract and ethyl acetate extract and chloroform extract and hexane extract	each 3.5 mg/cm^3^	each extract: r + on female adult *Anopheles stephensi* and *Culex quinquefasciatus*	ethanol extract: after 40, 80, 120, 160, 200 and 240 min: 100% repellency; for the other extracts the effect varied (e.g. chloroform extract: 120 min: 94.4.8 ± 1.81% repellency 160 min: 84.2 ± 2.38% repellency 200 min: 72.6 ± 2.6% repellency 240 min: 61.8 ± 2.48% repellency)
Barnard (1999)	Repellency test: number of mosquito bites counted at 30 min intervals; 2 replicates per treatment with two different humans	Human arm	Varying	***Thymus vulgaris***	Essential oil	5, 10, 25, 50, 75, and 100%	r + on female adult *Aedes aegypti* and *Anopheles albimanus* alone and in mixture with *Syzygium aromaticum*	5%: no repellent effect; 10%: no repellent effect 25%: *Aedes*: 45 min (±21); *Anopheles*: 45 min (±21) 50%: *Aedes*: 75 min (±21); *Anopheles*: 30 min (±0) 75%: *Aedes*: 75 min (±21); *Anopheles*: 60 min (±42) 100%: *Aedes*: 135 min (±21); *Anopheles*: 105 min (±21) 50% clove +50% thyme: *Aedes*: 150 min (±42) protection time; 75% clove +25% thyme: *Anopheles*: 135 min (±30) protection time = > The two mixtures were not significantly different
				*Thuja occidentalis*	Essential oil	5, 10, 25, 50, 75, and 100%	r0 on female adult *Aedes aegypti* and *Anopheles albimanus*	no repellent effect
Carroll et al. (2011)	Mosquito repellent essay on 4 × 8 cm only covered by a muslin cloth treated with different concentrations of EO, 6 replications	Human arm	1 min	***Juniperus communis***	Essential oil	0.750, 0.375, 0.187, 0.094, 0.047, 0.023, 0.011, 0.005 mg/cm^2^	r + on adult *Aedes aegypti*	minimum effective concentration = 0.057 ± 0.013 mg/cm^2^
Castillo‐Morales and Duque (2020)	Repellency bioassay (methodology of the American Society for Testing and Materials (ASTM))	Human arm	2 min and 15 min	***Salvia officinalis***	Essential oil	1000 mg/L	r + on adult female *Aedes aegypti*	Protection percentage (% ± SD): after 2 min: 67 ± 1 after 15 min: 26 ± 4.7
Choi et al. (2002)	Repellency bioassay (Rutledge 1994) + tests to determine protection time (Frances 1993)	5 hairless mice	Up to 60 min	***Lavandula angustifolia***	Essential oil	0.05%	r + on adult female *Culex pipiens pallens*	Protection rate: 65% Duration of protection (min): 31.0 ± 3.67
				***Rosmarinus officinalis***	Essential oil	0.05%	r + on adult female *Culex pipiens pallens*	Protection rate: 77% Duration of protection (min): 47.0 ± 8.28
				***Thymus vulgaris***	Essential oil	0.05%	r + on adult female *Culex pipiens pallens*	Protection rate: 91% Duration of protection (min): 65.4 ± 12.2
Drapeau et al. (2009)	y‐tube olfactometer 8 replications per stimulus, recording of mosquito responses	Human finger	30 sec	***Cupressus sempervirens***	Essential oil	0.01 mg/cm^2^	r + on adult females *Aedes aegypti*	Reduced activity of mosquitoes in olfactometer (around 60%) and reduced attractiveness of finger
				***Laurus nobilis***	Essential oil	0.1%, 0.3%, 1%, 3%, 10%	r + on adult females *Aedes aegypti*	Same as *Cupressus sempervirens*. No significant differences between the different concentrations
				***Rosmarinus officinalis***	Essential oil	1%	r + on adult females *Aedes aegypti*	Reduced activity of mosquitoes in olfactometer (around 70%) and reduced attractiveness of finger
				***Thymus vulgaris***	Essential oil	1%	r + on adult females *Aedes aegypti*	Reduced activity of mosquitoes in olfactometer (around 84%) and reduced attractiveness of finger
Giatropoulos et al. (2013)	Assessment based on human landing counts with 8 replicates per treatment	Human arm	5 min	***Cupressus sempervirens***	Essential oil	0.2 mg/cm^2^	r + on adult *Aedes albopictus*	Significant repellent effect: around 5 landings compared to 30 landings for the untreated control
				*Chamaecyparis lawsoniana*	Essential oil	0.2 mg/cm^2^, 0.08 mg/cm^2^	r + on adult *Aedes albopictus*	Complete repellence (0 landings) at the high concentration (0.2 mg/cm^2^) High repellence (1 landing) at the low concentration (0.08 mg/cm^2^) compared to 30 landings for the untreated control
				***Lavandula angustifolia***	Essential oil	0.2 mg/cm^2^, 0.08 mg/cm^2^	r + on adult *Aedes albopictus*	mean number of landings ± SEM after 5 min 0.2 mg/cm^2^: 0 landings 0.08 mg/cm^2^: 0.7 ± 0.3 landings
				***Rosmarinus officinalis***	Essential oil	0.2 mg/cm^2^, 0.08 mg/cm^3^	r + on adult *Aedes albopictus*	mean number of landings ± SEM after 5 min 0.2 mg/cm^2^: 0 landings 0.08 mg/cm^2^: 0 landings 0.04 mg/cm^2^: 17.9 ± 8.4 landings
				***Thymus vulgaris***	Essential oil	0.2 mg/cm^2^, 0.08 mg/cm4	r + on adult *Aedes albopictus*	mean number of landings ± SEM after 5 min 0.2 mg/cm^2^: 0 landings 0.08 mg/cm^2^: 13.7 ± 1.7 landings
Gillij et al. (2008)	Arm‐in‐cage assay: hand and arm protected by sleeve/glove except for 30 mm circle on forearm. each treatment tested once on 3 human subjects, randomized	Human hand and arm	Various from 0–90 min (until first bite)	***Rosmarinus officinalis***	Essential oil	90%, 50%, 25%, 12.5%	r + on adult female *Aedes aegypti*	mean repellence time: 90% EO: 90 min (max of this study) 50% EO: 90 min (max of this study) 25% EO: 80 ± 10 min 12.5% EO: 60 ± 17 min
Girgenti and Suss (2002)	Repellency bioassay	Human arm	For 3 min after 0 h, 1 h, 2 h and 3 h	***Lavandula angustifolia***	Cream and lotion (mixtures)	cream: 1 mg/cm^2^, lotion: 0.8 mg/cm^2^	cream: r0 on adult *Aedes aegypti*; lotion: r + on adult *Aedes aegypti*	No protection for cream; For lotion: Protection time: 0.8 ± 0.66 h → significantly different from control (*p* < 0.05)
				***Salvia officinalis***	Lotion (mixture)	0.8 mg/cm^2^	r + on adult *Aedes aegypti*	Protection time: 0.8 ± 0.66 h → significantly different from control (*p* < 0.05)
				***Thymus vulgaris***	Lotion (mixture)	0.8 mg/cm^3^	r0 on adult *Aedes aegypti*	Protection time: 0.4 ± 0.37 h → not significantly different from negative control (*p* < 0.05)
Govindarajan (2011)	Repellency bioassay (human‐bait technique)	Human arm	Up to 180 min	***Rosmarinus officinalis***	Essential oil	1.0, 2.5 and 5.0 mg/cm^2^	r + on adult female *Anopheles subpictus* and adult female *Culex tritaeniorhynchus*	1 mg/cm^2^: 100% repellency up to 30 min, then continuous decrease; 2.5 mg/cm^2^: 100% repellency up to 60 min, then continuous decrease; 5 mg/cm^2^: 100% repellency up to 90 min, then continuous decrease
Hieu et al. (2010)	Repellency bioassay: 5 × 5 cm opening in rubber glove, exposure 10 min after application for 5 min and then every 20 min until bite recieved. 3 replications per treatment	Human hand	5 min + various	***Lavandula angustifolia***	Essential oil	0.5 mg/cm^2^	r + on adult female *Stomoxys calcitrans*	Protection time (0.5 mg/cm^2^): 0.48 ± 0.015 h → weak repellency
				***Rosmarinus officinalis***	Essential oil	0.5 mg/cm^2^	r + on adult female *Stomoxys calcitrans*	Protection time (0.5 mg/cm^2^): 0.21 ± 0.024 h → weak repellency
				***Satureja montana***	Essential oil (pure and in mixture)	0.5 and 0.25 mg/cm^2^	r + on adult female *Stomoxys calcitrans*	Protection time (0.5 mg/cm^2^): data not available because skin irritation caused at this concentration Protection time (0.25 mg/cm^2^): 1.00 ± 0.069 h and for mixture: Protection time: 2.04 ± 0.029 h
				***Thymus vulgaris***	Essential oil (red thyme and white thyme)	0.5 and 0.25 mg/cm^2^	r + on adult female *Stomoxys calcitrans*	red thyme: Protection time (0.5 mg/cm^2^): 1.24 ± 0.039 h; (0.25 mg/cm^2^): (0.38 ± 0.024 h); white thyme: Protection time (0.5 mg/cm^2^): (2.12 ± 0.026 h); (0.25 mg/cm^2^): (0.58 ± 0.042 h)
Jebanesan et al. (2021)	Repellency bioassay, 5 replications per treatment	Human arm	Each 5 min after 40, 80, 120, 160, 200, 240 min	***Punica granatum***	Methanol extract and ethyl acetate extract	3.5 mg/cm^2^	both extracts: r + on adult female *Anopheles stephensi* and *Culex quinquefasciatus*	methanol extract: 100% repellency after 40 min, 80 min, 120 min, 160 min, 200 min and 240 min; ethyl acetat extract: 100% repellency after 40 min, 80 min, 120 min, 160 min and 200 min After 240 min: 97 ± 2.5% (*Anopheles*) and 93 ± 2.4% (*Culex*)
Koutsaviti et al. (2015)	Repellency assay based on number of mosquitoes landing on human skin treated with EO (two doses)	Human arm	5 min	*Pinus nigra*	Essential oil	0.2 μL/cm^2^ 0.4 μL/cm^2^	r + on adult *Aedes albopictus*	Between 2 and 14 landings at both concentrations and 3 origins of plant material compared to 51 landings in negative control.
			5 min	*Pinus strobus*	Essential oil	0.2 μL/cm^2^ 0.4 μL/cm^2^	r + on adult *Aedes albopictus*	9.5 landings at 0.2 μL/cm^2^ and 12 landings at 0.4 μL/cm^2^ compred to 51 landings in untreated control
Kulma et al. (2018)	Repellency bioassay, each repellent tested with 10 volunteers with 2 replications each.	Human arm	60 min	***Rosmarinus officinalis***	Leachate	2 mL pure	r0 on adult *Aedes aegypti*	no significant repellent effect → repellent efficacy lower than 10% after 10 min
			10 min, 30 min, 60 min	*Juglans regia*	Leachate	2 mL pure	r + on adult *Aedes aegypti*	After 10 min: 49 ± 19.2% repellency After 30 min: 34.3 ± 17% repellency After 60 min: 18.2 ± 17.4% repellency
Motevalli‐Haghi et al. (2020)	Repellency bioassay with shaved albino guinea pigs	Guinea pig	30 min	*Sambucus ebulus*	Methanol extracts and hexane extracts of fruit or leaves	50, 100, 150 mg/kg	r + on adult female *Culex pipiens*	methanol extract leaves: 30% repellency at 50 mg/kg, 80% repellency at 150 mg/kg; methanol extract fruit: 39% repellency at 50 mg/kg, 84% repellency at 150 mg/kg; hexane extract leaves: 14% repellency at 50 mg/kg, 67% repellency at 150 mg/kg; hexane extract fruit: 19% repellency at 50 mg/kg, 72% repellency at 150 mg/kg; 100 mg/kg always intermediate.
Sutthanont et al. (2022)	Repellency bioassay	Human arm and hand	Varying	***Rosmarinus officinalis***	Essential oil	100ul of pure essential oil	r + on adult female *Aedes aegypti*, *Anopheles dirus* and *Culex quinquefasciatus*	Protection time: 30 min (*Aedes aegypti* and *Anopheles dirus*); Protection time: 210 min (*Culex quinquefasciatus*)
				***Salvia officinalis***	Essential oil	100 μl of pure essential oil and mixtures 1:1 with several plants	r + on adult female *Aedes aegypti*, *Anopheles dirus* and *Culex quinquefasciatus*	Pure: Protection time: 150 min (*Aedes aegypti* and *Anopheles dirus*); Protection time: 300 min (*Culex quinquefasciatus*); mixtures: varying between protection time: 120–270 min
Traboulsi et al. (2005)	Human‐bait technique; number of mosquitoes bites and time until first bite was recorded. EO tested alone, with vaseline and with olive oil, 4 replications per treatment	Human arm	2 min up to 15 min	***Laurus nobilis***	Essential oil	1% and 3%	r + on adult *Culex pipiens molestus*	Protection time varied regarding formulation: EO alone: not significantly different from control; EO with vaseline: significantly differnt from control (1%: 18.12 min); EO with olive oil: significantly different from control (1%: 26.57 min; 3%: 52.37 min)
				***Pinus pinea***	Essential oil	1% and 3%	r + on adult *Culex pipiens molestus*	Protection time: EO alone: not significantly different from control; EO with vaseline: (1%: 1.07 min → unsignificant 3%: 18.35 min → significant) EO with olive oil: significant (1%: 18.02 min; 3%: 35.1 min)

Abbreviations: EO, essential oil; h, hours; min, minutes; r +, positive repellent effect (of different duration and strength, but significant for at least one concentration compared to the control arm/group/treatments); r0, zero repellent effect (not significantly different from control).

#### Outcome of outdoor trials

The systematic review process resulted in four outdoor studies (with 10 experiments). Cilek et al. (2011) investigated a product containing essential oil of *R. officinalis*. In a wire cage mosquito bioassay, the 45‐second application of the product on adult females of *A. albopictus* and *Cx. quinquefasciatus* had an adulticidal effect that varied depending on the distance (3 m: 9.6 ± 0.1%; 6 m: 13.1 ± 4.4%; 9 m: 3.3 ± 1.1%; 12 m: 6.4 ± 1.5%; 15 m: 5.5 ± 2.6%; 20 m: 0%). Applied as a single substance, the essential oil of *L. angustifolia* showed a repellent effect on adult tabanids (Diptera: Tabanidae) *Tabanus bromius* L. and a less intensive effect on *Tabanus tergestinus* Egger and *Haematopota pluvialis* L. in a trap test (tent‐like canopy traps baited with 4 mL of 1‐octen‐3‐ol (attractant) and with 16 mL of essential oil); this difference was not statistically significant (Krcmar & Gvozdic 2016a).

In glue traps, pure seed pots and fruits of *Ficus carica* L. (Rosales: Moraceae) and *P. granatum* were shown to have an attractive effect on adult *A. albopictus* (Müller et al., 2011). The smoke from *O. europaea* leaves caused an 80% reduction of indoor mosquito density (adult *Anopheles* spp.) compared with the control group in a 5.5‐hour trial in huts. The use of this smoke in a tent trial significantly inhibited the feeding activity of *Anopheles arabiensis* Patton (Diptera: Culicidae) on calves; 94.5% of mosquitoes remained unfed when exposed to the smoke of *O. europaea*, whereas in the control tents only 19.5% stayed unfed (Olbamo et al., 2021).

## DISCUSSION

The large number of publications and experiments provides a sound base of evidence‐based knowledge about the interaction of plants with blood‐feeding insects.

The vast majority (98%) of the experiments found were conducted under laboratory conditions, predominantly in vitro studies. These potentially provide important data on the mechanism of action of biologically active compounds and are therefore valuable in biomedical experiments. However, there often is a considerable discrepancy between in vitro and in vivo experiments due to the major challenge of extrapolation (R. Barnard & Gurevich, 2005). In the field of veterinary phytotherapy, it is apparent that systematic literature searches tend to find a far greater number of in vitro studies than in vivo or clinical trials. For example, a systematic review of medicinal plants in gastrointestinal and respiratory diseases in young livestock found 418 relevant references, of which only 48 references included clinical trials in veterinary medicine (Ayrle et al., 2016). In a systematic review on medicinal plants in canine dermatitis, out of 145 references, 100 references reported results of in vitro studies (Tresch et al., 2019). This is consistent with the results of our search with only 10 outdoor experiments and just two on a target host (cattle).

We integrated experiments applying mixtures in this systematic review in order to include as much information as possible about the plants from our list. Experiments testing mixtures accounted for approximately 15% of all experiments. Several publications included both experiments with mixtures and with single plant preparations (e.g. essential oils of *Pinus nigra* and *Satureja montana* tested alone or in a 1:1 mixture in (Pavela et al., 2018)); essential oils of *Rosmarinus officinalis* and *Salvia officinalis* tested alone or in combination with other plants by (Sutthanont et al., 2022). These cases show that results were similar if a plant was tested alone or in a mixture. Nevertheless, these results should be considered in more detail if plants from mixtures are to be investigated further.

In the experiments found in our systematic research, essential oils of plants were analysed most frequently (65%), followed by extracts (25%). Essential oils are extracted from aromatic plants with several methods of extraction, such as low‐ or high‐pressure distillation employing boiling water or hot steam. Essential oils are very complex natural mixtures that can contain around 20–60 constituents in very different concentrations, with usually two or three main constituents present in quite high concentrations (20%–70%) (Bakkali et al., 2008). The concentration of these substances, which make up the effect of essential oils, is much lower in fresh plant material than in extracts. For example, thyme herb contains 1%–2.5% essential oil, phenylpropane derivatives and flavonoids. Pure thyme essential oil contains 30%–50% thymol (Brendieck‐Worm & Melzig, 2018a).

Empirical knowledge of farmers in the context of pasture farming was collected in Switzerland (Bischoff et al., 2016; Disler et al., 2014; Holzner et al., 2024; Mayer et al., 2017; Mertenat et al., 2019; Schmid et al., 2012; Stucki et al., 2019) and the alpine foothills in Germany (Schlittenlacher et al., 2022). The comparison of these ethnoveterinary studies with the present systematic review revealed an overlap for five plant species, namely *Juglans regia* L. (Fagales: Juglandaceae), *L. nobilis, L. angustifolia, Picea abies* (L.) Karst (Pinales: Pinaceae) and *S. officinalis* (Table 5). Farmers mainly reported observations and experiences of animal behaviour under high insect pressure. For example, it was reported for *J. regia* that the flock of sheep always crowded under the tree on days with high insect pressure, whereas on other days, they favoured other trees and places on the pastures (Schlittenlacher et al., 2022). In the same study, there were a total of 18 use reports on the targeted application of those plant species in the environment or directly on the animals to repel flies (e.g. *M. domestica*, *L. sericata*). The use of some of these plants, for example *J. regia* as a fly repellent in cattle and horses (Mangold & Reicherter, 1907) or the use of *L. angustifolia* against insects in all domestic and farm animals can also be found in historical literature from the field of veterinary pharmacology and agricultural science (Fröhner, 1900).

**TABLE 5:** Comparison of the effects of the plant species covered by the systematic review on insecticidal, insect repellent and/or attractant properties of shrubs and trees growing in the alpine region on insects as reported by animal holders (ethnoveterinary use reports).

Plant species (botanical name)	Ethnovet‐erinary use report (UR)	Animal species	Application (app) or self‐selected behaviour (ssb)	Comment from animal holders regarding use	Total number of experiments (exp) and publications (pub) in systematic review	In vitro studies and study design	Studies with host or outdoor studies and study design
*Corylus avellana*	1 DE	Sheep	ssb	Repellence against flies, mosquitoes and horseflies	0	0	0
***Juglans regia***	5 DE, 5 CH^a^	3 UR for horse, each 2 UR for laying hen, sheep, each 1 UR for cattle, dog, goat	7 app, 2 app environment, 1 ssb	Repellence against flies and mites	5 exp. from 3 pub	4 exp/2 pub^1^: larvicidual assay on *Aedes aegypti*, *Anopheles stephensi* and *Culex quinquefasciatus*	1 exp/1 pub^2^: Repellency on adult *Aedes aegypti*; human host
***Laurus nobilis***	2 DE	Not specified	app	Repellence against flies, mosquitoes and horseflies	35 exp. from 13 pub	33 exp/12 pub^3^: Larvicidal activity, pupicidal activity, ovicidal activity, longevity, fecundity, Delayed effects (progeny sex ratio), repellency effect on *Aedes aegypti*, *Aedes albopictus, Anopheles stephensi, Culex pipiens, Culex quinquefasciatus, Culiseta longiareolata*	2 exp/2 pub^4^: Repellency on adult *Aedes aegypti* and *Culex pipiens molestus*; human host
***Lavandula angustifolia***	2 DE, 3 CH^b^	1 UR each for horse, donkey, cattle, 2 UR for not specified	3 app, 2 app environment	Repellence against flies	60 exp. from 22 pub	52 exp/20 pub^5^: ovicidal activity, oviposition deterrence, larvicidal activity, pupicidal activity, adulticidal activity, ingestion toxicity, repellency effect on *Aedes aegypti, Aedes albopictus, Culex pipiens, Forcipomyia taiwana, Haematopota pluvialis, Lucilia sericata, Musca domestica, Stomoxys calcitrans, Tabanus bromius, Tabanus tergestinus*	8 exp./5 pub^6^: Repellency effect against *Aedes aegypti, Culex pipiens pallens, Haematopota pluvialis, Tabanus tergestinus, Tabanus bromius*
***Picea abies***	1 CH^c^	Goat	app	Prophylactic repellence against flies	5 exp. from 2 pub	5 exp./2 pub^7^: larvicidal activity, adulticial activity, sublethal effects on vitality and on larval development of *Musca domestica* and *Culex quinquefasciatus*	0
***Salvia officinalis***	1 DE	Laying hen	app	Add to the laying nest to repel mites	29 exp. from 9 pub	17 exp/7 pub^8^: ovicidual activity, oviposition deterrence, pupicidal activity, larvicidal activity, DNA damage, mitochondrial alterations, Acetylcholinersterase inhibition, Repellency effect on *Aedes aegypti, Anopheles quadrimaculatus, Culex pipiens, Culex quinquefasciatus*	12 exp./3 pub^9^: Repellency effect on *Aedes aegypti, Anopheles dirus, Culex quinquefasciatus*, human host

Abbreviations: app, applications; CH, Swiss ethnoveterinary UR from different cantons; DE, German ethnoveterinary UR from Bavaria (Schlittenlacher et al., 2022); exp., experiment(s); pub, publication(s); sbb, self‐selected behavior; UR, use report.

^a^ (Disler et al., 2014; Mayer et al., 2017; Mertenat et al., 2019; Schmid et al., 2012).

^b^ (Disler et al., 2014; Mayer et al., 2017; Mertenat et al., 2019).

^c^ (Bischoff et al., 2016).

^1^ (Ali et al., 2019; Köroǧlu et al., 2016).

^2^ (Kulma et al., 2018).

^3^ (Andrade‐Ochoa et al., 2018; Bouzidi et al., 2020; Erler et al., 2006; Fernandez et al., 2018; Pavela, 2009; Sheng et al., 2020; Tabanca et al., 2013; Traboulsi et al., 2005; Wu et al., 2022; Zarenezhad et al., 2022).

^4^ (Drapeau et al., 2009; Traboulsi et al., 2005).

^5^ (Adams et al., 2016; Ahmadi et al., 2022; Al‐Massarani et al., 2019; Amer & Mehlhorn, 2006a; Bedini et al. 2019; Bosly & Bosly, 2022; Conti et al., 2010; El‐Akhal et al., 2021; Giatropoulos et al., 2018; Guo, Luo, et al., 2023; Hazarika et al., 2020; Kang et al., 2009; Khater et al., 2011; Pavela, 2007a, 2009; Sajfrtova et al., 2013; Sheng et al., 2020; Sinthusiri & Soonwera, 2013, 2014; Wu et al. 2013).

^6^ (Choi et al., 2002; Giatropoulos et al., 2018; Girgenti & Suss, 2002; Hieu et al., 2010; Jaenson et al., 2006; Krcmar & Gvozdic, 2016).

^7^ (Levchenko et al., 2021; Pavela et al., 2021).

^8^ (Ali et al., 2015; Baz et al., 2022; Castillo‐Morales et al., 2019; Castillo‐Morales & Duque, 2020; Kang et al., 2009; Pavela, 2007a; Rios et al., 2017).

^9^ (Castillo‐Morales & Duque, 2020; Girgenti & Suss, 2002; Sutthanont et al., 2022).

The perennial woody plants most frequently investigated in the studies identified by the systematic review (*T. vulgaris, R. officinalis, L. angustifolia, S. officinalis*) are well known for their high concentration of essential oil and for their distinctive odour. They are very aromatic and are therefore also known as culinary herbs and spices (Stefanaki & van Andel, 2021). These plants could benefit animal health due to their positive effect on the gastrointestinal and respiratory tract systems, for example (Brendieck‐Worm & Melzig, 2018b). However, in view of the practicality of using them for grazing animals in the context of agroforestry, it is questionable whether they are suitable for cultivation on pastures. Due to their small growth habit, they are less useful for insect protection and are presumably much less effective as a fresh plant than essential oils. Nevertheless, such aromatic plants may serve in specific formulations to be applied to the animals.

Larger trees and shrubs are an essential component of biodiversity. They are used by grazing animals (e.g. cattle and horses) as a natural additional source of food, for comfort behaviour and as a shelter (Popp & Scheibe, 2013). Some larger shrubs and trees may be considered for integration into agroforestry systems with a benefit for biodiversity and for the grazing animals. Two of them, the pomegranate and olive, mainly grow on the southern edge of the Alps, but may be cultivated in more northern regions as well. A total of 58 experiments were carried out on the pomegranate, *P. granatum* (14 experiments with fruit, 44 with leaves). They showed repellent, larvicidal, ovicidal and adulticidal effects. *P. granatum* was cultivated in southern Switzerland in the days of the Roman Empire (Jacomet et al., 2002), but today it plays a minor role in modern agroforestry and is not necessarily an optimal tree for Swiss pastures, especially depending on the region and altitude. The effect of smoke and extracts of leaves (nine experiments), oil (three experiments) and essential oil of fruit (two experiments) of *O. europaea* (olive) was investigated in five publications. However, it was only tested on *A. aegypti* and *A. arabiensis*, which are of little relevance to grazing animals in Switzerland. Furthermore, the ‘exotic’ nature of the tree for large areas of Switzerland must also be considered here. In Switzerland, *O. europaea* is mainly cultivated in the canton of Ticino (FondazioneDiamante, 2020).

Other tree and shrub species are present in the whole alpine region as well as in the more northern parts of Switzerland. They may be more suitable for agroforestry systems for their easy cultivation and inexpensiveness. *A. glutinosa* (common alder), *J. communis* (common juniper), *J. regia* (common walnut) and *P. abies* (Norway or European spruce) are trees that grow naturally in meadows and forests in large parts of Switzerland (Infoflora, 2015). They are easy and inexpensive to cultivate and are fast‐growing. The experience reports of Swiss and German farmers on *J. regia* and *P. abies* indicate that grazing animals like to seek out these trees and tend not to avoid them (Table 5). Furthermore, the plant parts of these four trees are non‐toxic or slightly toxic (*J. communis* fruit; CliniPharm/CliniTox, 2024), therefore no harm to grazing animals is to be expected.

*Alnus glutinosa* had a larvicidal effect on 3rd instar larvae of *Cx. pipiens* (David et al., 2000), which is widespread in Switzerland, and plays a crucial role in the transmission of viruses in Europe (especially medical and veterinary arboviruses; Brugman et al., 2018).

*Juniperus communis* was tested in several experiments against *A. aegypti, A. albopictus, A. stephensi* and *Cx. quinquefasciatus* and had repellent, larvicidal and adulticidal effects against these species (Supplementary File 2). The essential oil also had a repellent effect of 73.3% against the tick *Ixodes scapularis* Say (Ixodida: Ixodidae; Carroll et al., 2011). Furthermore, Levchenko et al. (2021) demonstrate an adulticidal effect of the essential oil on *M. domestica*, which was still >60% at a 1:500 dilution. In another publication, the adulticidal effect of the male and female plant was compared and an adulticidal effect of both plant sexes on both fly sexes was demonstrated (Pavela et al., 2021).

*Juglans regia* had a repellent and larvicidal effect on *A. aegypti* in the laboratory (Köroǧlu et al., 2016; Kulma et al., 2018). In the ethnoveterinary reports from Switzerland and Germany, a total of 10 farmers stated a repellent effect against native flies and mites (Table 5). Austrian farmers have also reported hanging bunches of *J. regia* in the stable to scent the air and hinder the development of flies (Vogl et al., 2016). In addition to a possible effect on ectoparasites, the leaves of *J. regia* have also been described as having an antiparasitic effect on internal parasites such as gastrointestinal helminths, for example in small ruminants (Mir et al., 2024).

*Picea abies* essential oil had larvicidal effects on the third larval stage of *Cx. quinquefasciatus* (Pavela et al., 2021) and adulticidal activity on *M. domestica* (Levchenko et al., 2021; Pavela et al., 2021). *M. domestica* carries and transmits a large number of pathogens (viruses, bacteria, fungi and parasites) that can cause serious diseases in humans and animals (Khamesipour et al., 2018) and interfere with the behaviour and performance of farm animals, such as dairy cows (Renčínová et al., 2021). Natural protection options for our livestock are therefore needed.

The results of this systematic review can inform future research. However, it is important to note that reports on in vivo experiments with target species are scarce, and challenges can arise when transitioning from bioassays to field studies. For example, Eastern red cedar *Juniperus virginiana* L. (Cupressales: Cupressaceae) has demonstrated high potential for pest control in laboratory studies and an outdoor bioassay due to its toxicity and repellency to arthropods (Eller et al., 2014). By contrast, medically important mosquitoes were found in significantly higher numbers in areas with more Eastern red cedars (Noden et al., 2021). Furthermore, when considering the effects of repellents, it should be noted that the effects of contact repellents may not overlap with the effects of spatial repellents. In particular, the concentrations of plant‐derived compounds reported in in vitro studies are difficult to extrapolate to field conditions with respect to the spatial repellent effect. Effective exposure would require grazing animals to remain in close and continuous proximity to the relevant trees or shrubs. Contact repellents may be more useful when applied topically to host animals, whereas the spatial repellent effect is presumably more important when selecting tree and shrub species to provide natural shelter for grazing animals in agroforestry systems. Therefore, a literature search should investigate the available information on the repellent and toxic effects of the promising plants identified in this systematic review. Based on these results, studies involving livestock should initially be conducted under semi‐controlled conditions, progressing to studies with grazing livestock that are either treated topically with contact repellents or exposed to spatial repellents.

The systematic survey covered many insect species of medical importance, including numerous mosquito species. However, some species of imminent veterinary importance, such as horn flies (Sciomyzidae) and blow fly (Calliphoridae) species other than *L. sericata* were not covered by the available studies. Future studies should address these species as well. Ideally, these studies as well should be field studies involving grazing animals in order to address the challenges of transitioning from bioassays to field studies and from plant preparations to whole plants.

Designing studies involving grazing animals in a natural environment with unknown insect populations presents a challenge in terms of species diversity. The defensive behaviours (e.g. head shaking, tail flicks and leg stamps) of hosts differ between individual animals and according to insect species and density (Hansen et al., 2023). Insect densities are difficult to estimate objectively and accurately, even if only one insect species is involved (Smythe et al., 2020). To obtain meaningful results, field studies investigating the effectiveness of shrubs and trees as a natural form of insect protection for grazing animals must therefore overcome these challenges and determine the composition and abundance of the natural insect population.

## CONCLUSION

The systematic review revealed a considerable number of studies with and without hosts (mainly in the laboratory) on bushes or woody shrubs, in particular aromatic herbs, that grow in many regions of Switzerland: thyme (*T. vulgaris*), rosemary (*R. officinalis*), lavender (*L. angustifolia*), and sage (*S. officinalis*). The trees most frequently found in the literature search (common alder *A. glutinosa*, pomegranate *P. granatum*, bay laurel *L. nobilis*, stone pine *P. pinea*, olive *O. europaea*) can only be partially cultivated in warmer regions of Switzerland; in the context of global warming, however, their cultivation could also become of interest in other regions. Publications were also found on common juniper *J. communis*, the European spruce *P. abies*, and the common walnut *J. regia*; Swiss farmers have anecdotally described these plants as insect and mite repellents. In the reported studies, they had significant repellent and larvicidal effects against mosquitoes and flies commonly found on pastures in Switzerland (common house mosquito *Cx. pipiens*, common house fly *M. domestica*, common green bottle fly *L. sericata*) as well as against tropical and sub‐tropical mosquitoes (*A. aegypti, Cx. quinquefasciatus*, *A. albopictus*), which also are found more and more frequently in Europe and Switzerland (e.g. in parts of the Rhine valley, around Lake Constance and Lake Neuchâtel in Switzerland).

The findings for several plant species, especially good cultivable ones like *A. glutinosa, J. communis, P. abies* and *J. regia* are promising. However, there are substantial differences between laboratory and outdoor conditions, and between the content of, for example, repellent or insecticidal active substances in plant preparations and in the whole plant. But this systematic review provides interesting information on trees and shrubs that are worth investigating in practice. The few outdoor studies found, especially those with cattle as the target species, reveal a potential that should be further explored. The information obtained can build a basis for trials with the most promising woody shrubs and trees on pastures during the summer months against blood‐feeding insects.

## AUTHOR CONTRIBUTIONS

**Theresa Schlittenlacher**: Conceptualisation; formal analysis; investigation; methodology; supervision; visualisation; writing—original draft preparation. **Sofie Egli**: Data curation; formal analysis; investigation. **Michael Walkenhorst**: Conceptualization; validation; writing—review and editing. **Veronika Maurer**: Conceptualisation; funding acquisition; formal analysis; investigation; project administration; supervision; writing—review and editing.

## FUNDING INFORMATION

This work was supported by Fondation Sur‐la‐Croix, Basel, Switzerland.

## CONFLICT OF INTEREST STATEMENT

The authors declare no conflicts of interest.

## Supporting information


**Supplementary File 1.** Preselection of plant species (woody and perennial shrubs, bushes and trees) that are native or cultivable in Switzerland.
**Supplementary File 2.** Description and outcome of the 503 experiments from the 114 publications in our systematic literature search on the pre‐searched tree and shrub species in combination with Diptera of veterinary importance.

## Data Availability

The data that support the findings of this study are openly available in Dryad at https://doi.org/10.5061/dryad.8w9ghx41c (Schlittenlacher et al. 2025).
